# Telehealth challenges during COVID-19 as reported by primary healthcare physicians in Quebec and Massachusetts

**DOI:** 10.1186/s12875-021-01543-4

**Published:** 2021-09-26

**Authors:** Mylaine Breton, Erin E. Sullivan, Nadia Deville-Stoetzel, Danielle McKinstry, Matthew DePuccio, Abi Sriharan, Véronique Deslauriers, Anson Dong, Ann Scheck McAlearney

**Affiliations:** 1 Department of Community Health Sciences, Université de Sherbrooke, 150, place Charles-LeMoyne, Room 200, Longueuil, QC J4K 0A8 Canada; 2 Healthcare Management, Sawyer School of Business, Suffolk University, Boston, USA; 3 Department of Global Health and Social Medicine/Center for Primary Care, Harvard Medical School, Boston, USA; 4 Department of Health Systems Management, Rush University, College of Health Sciences, Chicago, USA; 5 Institute of Health Policy, Management and Evaluation, University of Toronto, Toronto, Canada; 6 Mount Sinai Hospital Academic Family Health Team, Toronto, Canada; 7 Department of Family and Community Medicine and Center for the Advancement of Team Science, Analytics, and Systems Thinking (CATALYST), Ohio State University, Columbus, USA

**Keywords:** Telehealth, COVID-19, Primary healthcare, Family physicians

## Abstract

**Background:**

The COVID-19 pandemic has driven primary healthcare (PHC) providers to use telehealth as an alternative to traditional face-to-face consultations. Providing telehealth that meets the needs of patients in a pandemic has presented many challenges for PHC providers. The aim of this study was to describe the positive and negative implications of using telehealth in one Canadian (Quebec) and one American (Massachusetts) PHC setting during the COVID-19 pandemic as reported by physicians.

**Methods:**

We conducted 42 individual semi-structured video interviews with physicians in Quebec (N = 20) and Massachusetts (N = 22) in 2020. Topics covered included their practice history, changes brought by the COVID-19 pandemic, and the advantages and challenges of telehealth. An inductive and deductive thematic analysis was carried out to identify implications of delivering care via telehealth.

**Results:**

Four key themes were identified, each with positive and negative implications: 1) access for patients; 2) efficiency of care delivery; 3) professional impacts; and 4) relational dimensions of care. For patients’ access, positive implications referred to increased availability of services; negative implications involved barriers due to difficulties with access to and use of technologies. Positive implications for efficiency were related to improved follow-up care; negative implications involved difficulties in diagnosing in the absence of direct physical examination and non-verbal cues. For professional impacts, positive implications were related to flexibility (teleworking, more availability for patients) and reimbursement, while negative implications were related to technological limitations experienced by both patients and practitioners. For relational dimensions, positive implications included improved communication, as patients were more at ease at home, and the possibility of gathering information from what could be seen of the patient’s environment; negative implications were related to concerns around maintaining the therapeutic relationship and changes in patients’ engagement and expectations.

**Conclusion:**

Ensuring that health services provision meets patients’ needs at all times calls for flexibility in care delivery modalities, role shifting to adapt to virtual care, sustained relationships with patients, and interprofessional collaboration. To succeed, these efforts require guidelines and training, as well as careful attention to technological barriers and interpersonal relationship needs.

**Supplementary Information:**

The online version contains supplementary material available at 10.1186/s12875-021-01543-4.

## Background

The COVID-19 pandemic has revealed the critical importance of being able to provide effective telehealth that meets patients’ needs while reducing the risk of infection from SARS-CoV-2 [1–4]. The rapid transition to telehealth by primary healthcare (PHC) providers has showcased this care delivery modality as an alternative to traditional face-to-face patient consultations [5, 6]. Telehealth is defined as synchronous or asynchronous consultation using information and communication technologies such as telephone, video conferencing, or secure messaging [7] to overcome geographical and functional distance [8].

Since the beginning of the pandemic, telehealth has allowed remote triage of patients, rapid access to information, routine follow-up care (especially relevant for managing chronic conditions), remote diagnosis, and remote care of patients [2, 5]. This care delivery approach has helped reduce demand for emergency services and has improved access for some patients [9, 10]. Studies have identified other advantages, such as convenience, cost savings, and ease of organizing multidisciplinary visits and consulting colleagues in real time. However, research has also highlighted the necessity of preserving meaningful teamwork [4, 11, 12].

To date, the identified disadvantages of telehealth concern potential weakening of therapeutic relationships and decreased continuity of care, as well as lack of psychosocial support and depersonalization of practice [4, 13, 14]. Other identified disadvantages of telehealth include the risk of compromised confidentiality, as well as patients’ unequal access to and capacity for using technology, such that certain populations risk being excluded from this type of care, such as elderly [4, 11, 13] and vulnerable populations (e.g., persons living in rural areas or with low income, ethnic minorities, allophones, etc.) [15–17]. Another problem with telehealth is the inability to conduct direct physical examinations [4]. Finally, issues have been raised regarding the compatibility of certain professional activities with telehealth, as well as issues around interprofessional collaboration (workload, isolation, lack of socialization time) [12].

The imperative of providing telehealth that meets the needs of patients in a pandemic has raised many challenges for PHC providers. Its rapid implementation has raised questions about the implications of its use in different clinical contexts. Physicians adopted telehealth expeditiously because of the COVID-19 pandemic, but information on physician perspectives about telehealth is scarce [18]. To our knowledge, few studies have explored the perceptions of physicians about the rapid implementation of telehealth in the context of the COVID-19 pandemic.

### Context of the study: comparison between Massachusetts and Quebec

Prior to the COVID-19 outbreak, telehealth implementation had been, in most countries, very limited [2]. According to a 2019 Commonwealth Fund survey, 79% of physicians in the United States reported interacting online with their patients, compared to 23% in Canada [19]. Few PHC physicians in either Canada (16%) or the US (20%) reported using video consultations with patients before the COVID-19 pandemic [19]. Prior to the pandemic, only 17% of Quebec physicians reported using telehealth and only 3% reported using video consultations [19].

In both contexts, physicians rapidly implemented telehealth during the first months of the pandemic. In Massachusetts, during March and April 2020, the high uptake of telehealth accounted for two-thirds of PHC visits [20]. By April 2020, nearly half (43.5%) of Medicare PHC visits were conducted via telehealth. Additional data from community health centers in Massachusetts showed that, from January to April 2020, total telehealth visits for medical services rose from 506 to more than 83,000 [21]. In Quebec, during the same period, more than 80% of physicians practicing in university-affiliated family medicine groups reported conducting telephone consultations, while less than 3% conducted video consultations [22].

This whirlwind speed of change raises questions about how PHC physicians are adapting their practices to deliver care via telehealth. The aim of this study was to describe the positive and negative implications of using telehealth during the pandemic, as reported by physicians in one Canadian (Quebec) and one American (Massachusetts) primary healthcare setting, to help inform strategies to support the use of telehealth in primary healthcare across settings.

## Methods

### Design

We conducted a comparative qualitative study on PHC physicians’ perceptions about telehealth use in Quebec and in Massachusetts. Those two contexts are of interest, in that they present similarities with respect to limited telehealth implementation prior to the pandemic and differences in their health systems’ funding and regulation. We conducted individual semi-structured video interviews with physicians in Quebec and Massachusetts between September and December 2020.

### Data collection

Physicians were invited to participate in the study through recruitment emails and posts on social media, as well as through the newsletters of the Harvard Medical School Center for Primary Care and of family physician groups in Quebec. A snowball strategy was also used, in which participants identified other potential participants for the study [23]. Purposive sampling was done to balance physician characteristics in terms of gender, number of years of practice, and type of practice.

The research team developed a semi-structured interview guide (see Additional file 1) that covered three main topics: 1) practice history; 2) changes in practice brought about by the COVID-19 pandemic; and 3) advantages and challenges of telehealth observed during the pandemic. The interviews lasted between 30 and 65 minutes and were audio recorded using Zoom. Recordings were transcribed verbatim either by a secretary or using Zoom’s transcription feature, with the content subsequently reviewed and validated. Participants provided verbal or written consent prior to being interviewed. All transcripts were anonymized for analysis. Physicians were not compensated for their participation.

### Analysis

Using NVivo12 and guided by the principle of content saturation [24], we carried out an inductive and deductive thematic analysis [25] to identify both positive and negative implications of conducting telehealth during a pandemic. Our initial codebook was developed based on the interview guide. As the analysis progressed, several codes and categories were added inductively. MB, VD, and NDS piloted the initial codebook on three transcripts, discussing codes and themes after each interview and iteratively modifying the codebook in collaboration with ES and DM. To ensure the quality of the analysis, we regularly reviewed the coding, discussing emerging themes and their conceptual meanings. MB led all the interviews in Quebec and participated in almost all in Massachusetts, which were led by ES. The same codebook was used for the analysis of both sites’ data, each carried out by a person specialized in that context. Integrating the findings from both sites helped to identify themes related to positive and negative implications of using telehealth in a pandemic.

## Results

Forty-two physicians participated in the study: 20 family physicians in Quebec and 22 PHC physicians in Massachusetts, who provided care to patients in general internal medicine, pediatric, and family medicine practices. We identified four key themes related to conducting telehealth in PHC, as perceived by physicians in the two contexts: 1) access for patients; 2) efficiency of care delivery; 3) professional impacts; and 4) relational dimensions of care. For each theme, we report the positive and negative implications from those physicians’ perspectives (see Table 1).

**Table 1:**
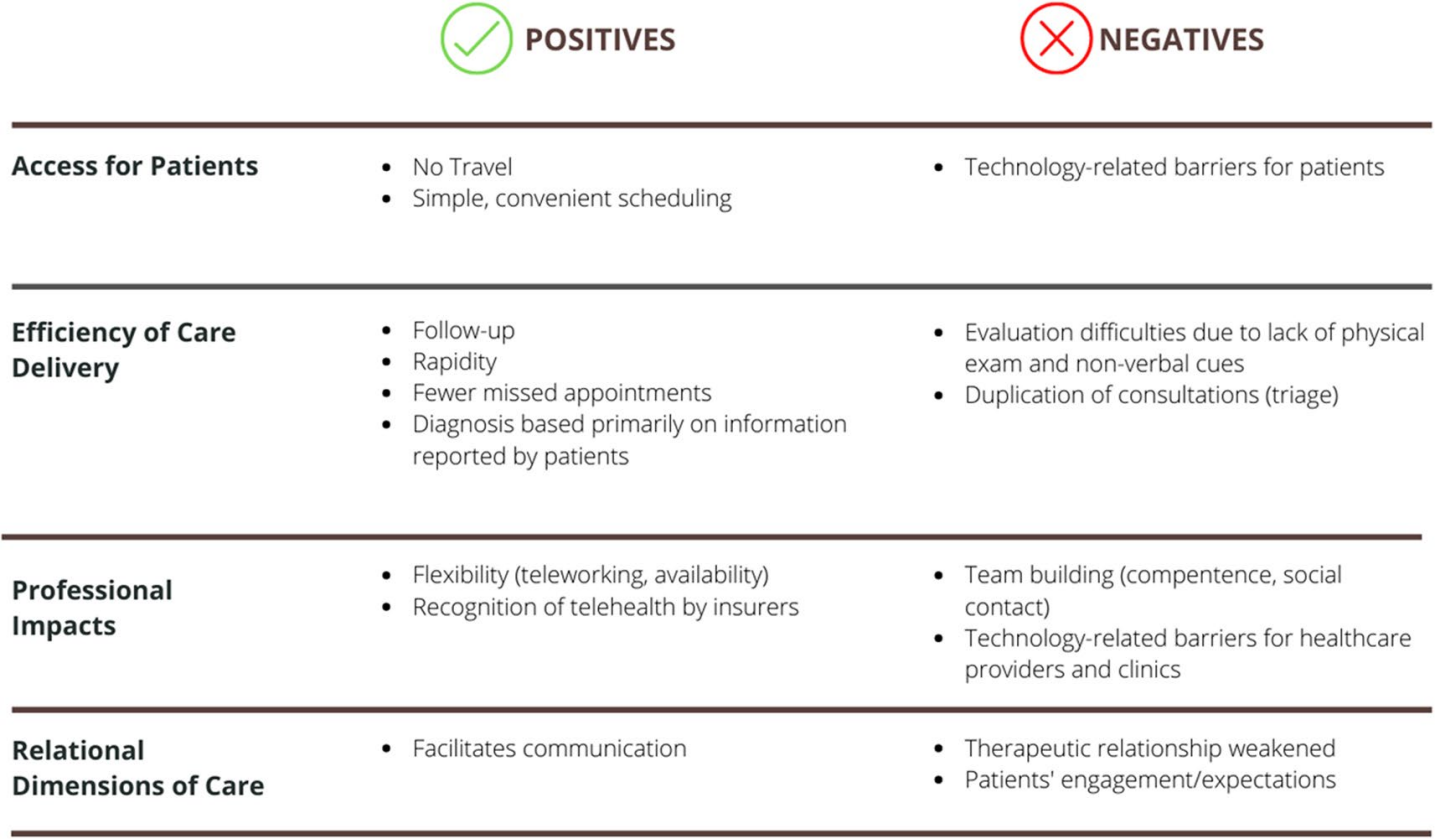
Key themes related to conducting telehealth during COVID-19

### Positive and negative implications related to patients’ access to health services

The first theme centered on the fact that accessing healthcare services via telehealth is easier and more convenient for some patients than face-to-face appointments. Patients do not need to travel to and from their appointments, take as much (or any) time off work, or spend time in waiting rooms. Not needing to travel was perceived as an advantage, in that this saves time as well as transit and parking costs. This was considered particularly beneficial for patients who experience physical or financial barriers to access, including the elderly, those with mobility impairments, those living in rural areas, and those with low incomes. Scheduling appointments was also perceived to be more convenient. According to respondents from both sites, patients appreciated not having to leave work for appointments or arrange for childcare. Our analyses suggest that physicians favor maintaining telehealth services following the pandemic, given that patients do not need to rearrange their schedules to the same extent as for face-to-face visits.

On the other hand, some patients face technological barriers that hinder their use of telehealth and thus their access to health services. For instance, video consultations presented difficulties for some patients, such that reverting to telephone (audio only) appointments was sometimes necessary. Respondents mostly perceived this as being due to patients’ lacking the necessary tools (e.g., email, smartphone), or the skills to adequately use these tools, as well as to characteristics such as hearing impairment or vulnerability (e.g., low income). Some Quebec respondents explained that they did not encourage their elderly patients to try the video telehealth experience. They considered that installing video capability could be complicated, time-consuming, and require technical support— difficult to obtain in a pandemic context—and thus did not focus on this care delivery modality for these patients. Table 2 presents quotes to support each sub-theme identified.

**Table 2:**
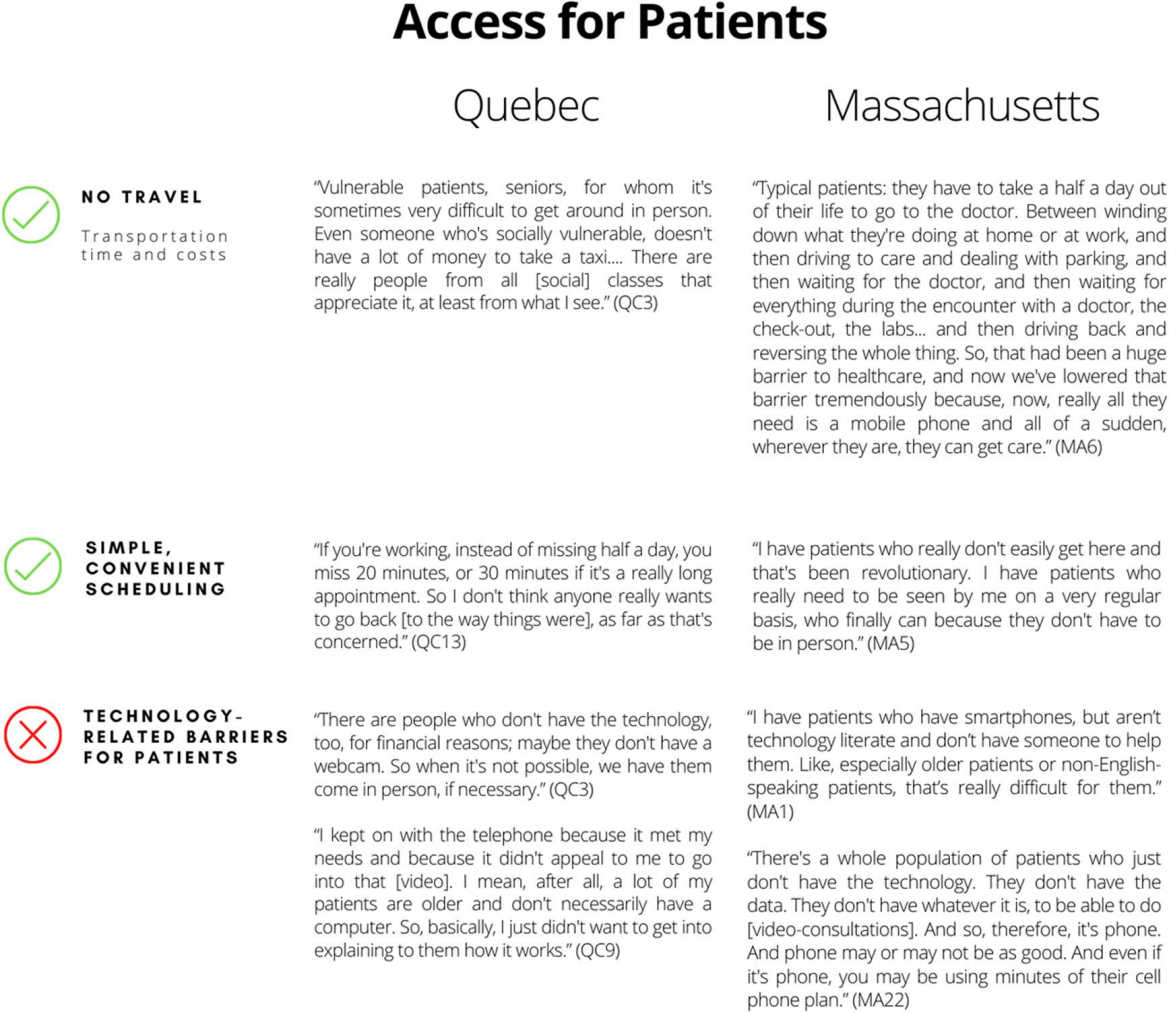
Positive and negative implications related to patients’ telehealth access

### Positive and negative implications related to efficiency of care delivery

The second key theme had to do with participants’ perceptions of how telehealth use transformed care delivery. Positive implications reported by respondents in Quebec and Massachusetts included increased efficiency for follow-up care, the ability to see patients more frequently when needed, improved rapidity of care delivery (shorter wait times for appointments, shorter duration of consultation), and fewer missed appointments. With respect to the diagnostic process, however, respondents had opposing perceptions about the effectiveness of remote consultations. On one hand, they now questioned the need for face-to-face appointments for situations in which the patient’s history was sufficient to make a diagnosis (i.e., no physical examination needed). On the other hand, they pointed out the difficulty of diagnosing without a physical exam and visual information. Table 3 presents key quotes from both care contexts.

**Table 3:**
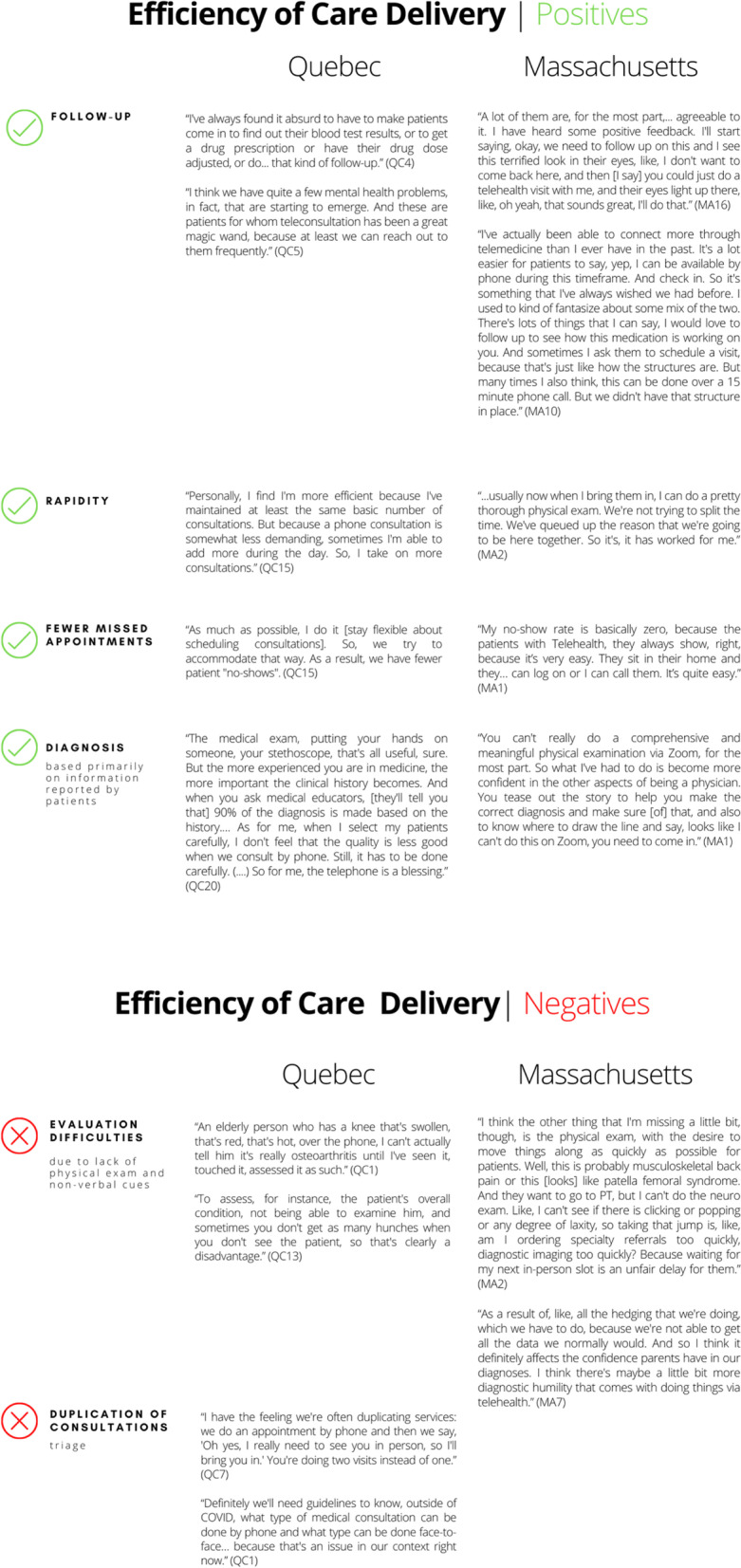
Positive and negative implications related to efficiency of care delivery

Participants considered telehealth to be excellent for follow-up appointments that did not require examinations, such as brief appointments focused on treatment compliance, the benefits or side effects of a new medication, or follow-up about mental health concerns. Also, telehealth reportedly made it possible for providers to connect more frequently with their patients, as needed.

Several physicians in both sites reported that telehealth appointments were sometimes less time-consuming than face-to-face visits. With respect to completing clerical work, telehealth appeared to have had a positive effect in Quebec, but both positive and negative effects were reported in Massachusetts. Some respondents reported they were better able to complete their notes when using telehealth, while others said they had trouble navigating video consultations and EMRs, resulting in their falling behind with notes and follow-up actions needed after the telehealth appointments. Another aspect of telehealth that contributed to perceptions of greater efficiency was the ability to communicate with patients via email to exchange documents, such as photographs. In Quebec, some physicians reported that the pandemic had accelerated their use of emails with patients, which they had not used before. Our results also suggest that telehealth may have increased efficiency by decreasing the number of missed appointments. This may have been because consulting remotely allowed more flexibility with appointment times and greater convenience for patients.

In terms of negative aspects related to efficiency of care delivery, physicians from both Quebec and Massachusetts reported that, for some pathologies, it is harder to establish a diagnosis without a physical exam and non-verbal information, making remote examination difficult. For example, some acute mental health and pain cases reportedly require face-to-face appointments, as do new musculoskeletal cases and pregnancy.

Respondents also raised concerns about potential medical errors, as telehealth made it more difficult to properly diagnose patients. For appointments conducted over the telephone, our results suggest that the lack of visual information hindered physicians’ ability to evaluate patients’ understanding of their condition (literacy, language barrier, difficulty in asking/responding to questions, etc.), thus posing diagnostic difficulties.

In Quebec, duplication of visits was mentioned as an important challenge. When remote consultation was not sufficient to assess patients’ conditions, sometimes patients had to come in for face-to-face visits. Our analyses revealed the importance of being able to assess beforehand the appropriateness of a telehealth consultation, as opposed to a face-to-face visit, when booking appointments. As nurses were redeployed from PHC to hospital settings during the COVID-19 pandemic, triage fell to administrative assistants, who lacked the necessary clinical training, and ultimately some physicians became involved in this role. In Massachusetts, this “duplication” of roles was perceived as a viable triage mechanism. Respondents from both sites reported that, in some practices, a telehealth appointment was required before a patient could be scheduled for a face-to-face consultation.

### Positive and negative implications related to professional impacts

This theme refers to how telehealth transformed the way providers work and to its impacts on physicians’ practices. The positive aspects in both contexts related to how teleworking had increased providers’ scheduling flexibility and availability for patients via telehealth appointments. The negative aspects related to decreased opportunities for team building and technological limitations. Table 4 shows keys quotes related to positive and negative professional impacts in both contexts.

**Table 4:**
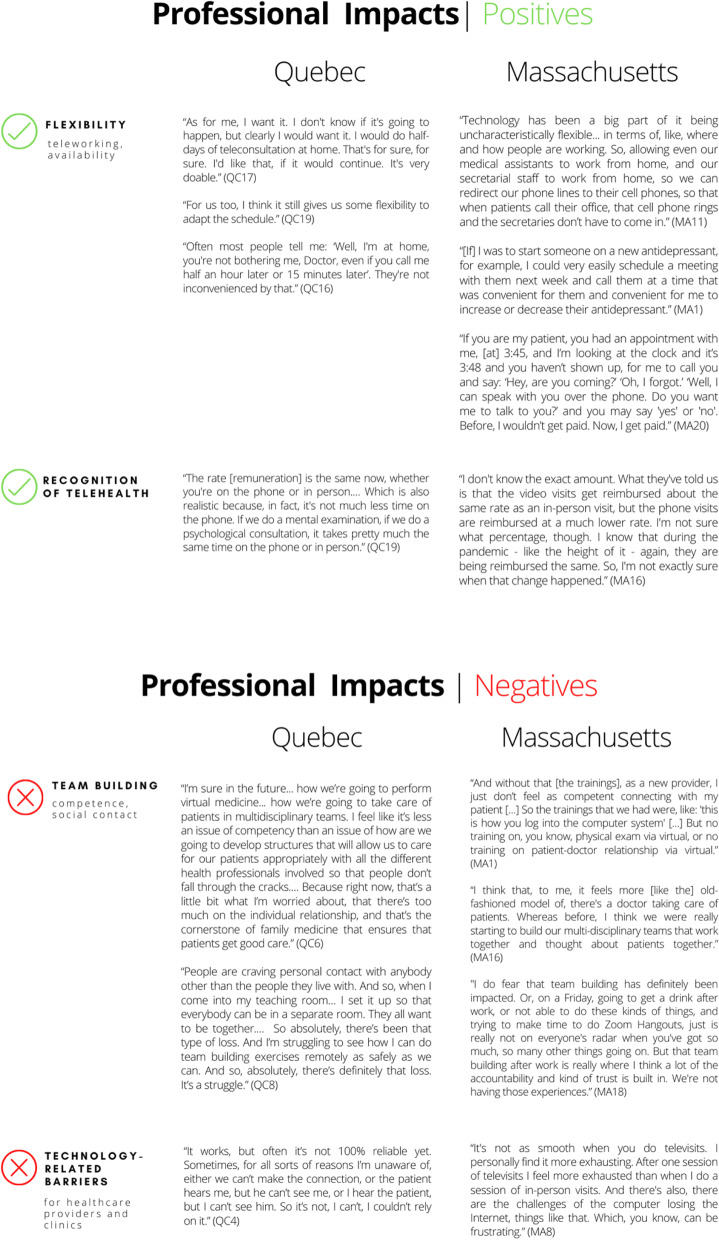
Positive and negative implications related to professional impacts

Physicians perceived that the ability to work from home improved their quality of life. Given the pandemic context, teleworking allowed those with a greater risk of contracting the virus (e.g., older physicians) to continue working, and several respondents emphasized their hope that teleworking remain possible in certain situations following the pandemic.

Telehealth also provided physicians greater scheduling flexibility and availability for patients. They could choose when they would be available for telehealth appointments. However, while physicians were less concerned about inconveniencing patients when they were delayed, given that those patients were not waiting in physical waiting rooms, their inability to notify patients about such delays was mentioned as a concern.

Prior to the pandemic, while some physicians provided telehealth services, the lack of compensation for these was a barrier to their use. Our respondents reported appreciating that telehealth is now formally recognized and reimbursed as a care provision modality.

The negative aspects reported by both Quebec and Massachusetts respondents related to team building (less sharing of competencies, reduced social contact) and technological limitations for providers and their practices. Because physicians are trained primarily to deliver care in person, our respondents considered the absence of telehealth training problematic, particularly with respect to using virtual tools to make diagnoses as well as the complexity of learning new software under pressure at the outset of the pandemic. Physicians also reported struggling with how to nurture and develop the patient–physician relationship remotely, particularly with new patients. Another reported drawback was reduced interactions among professionals, which had a negative impact on team building and hindered discussions of complicated cases.

Particularly in Quebec, challenges with video consultations were experienced due to lack of equipment (e.g., webcam) or insufficient internet bandwidth in some office settings that resulted in poor audio-visual quality. In Massachusetts, while this was not reported as a barrier for physicians, some said it had been a barrier for their patients. The effort required to install or set up video devices complicated the use of telehealth. Installing an application or looking for equipment needed to obtain care via telehealth were specifically mentioned as challenges for patients. In Massachusetts, some respondents said they had developed “workarounds” that were easier to use than the telehealth systems provided by their institutions. The most common workaround mentioned for when a patient could not connect for a video visit was simply switching to a telephone call and using an app on the physician’s telephone to disguise their personal number. A number of respondents noted that, while this was not the health system’s preference, it was easiest for both physicians and patients.

### Positive and negative implications related to relational dimensions of care

This theme addresses the challenges related to the relational aspects of medical telehealth practice. Table 5 presents quotes from interviews in both sites that support each subtheme associated with the relational dimensions of care.

**Table 5:**
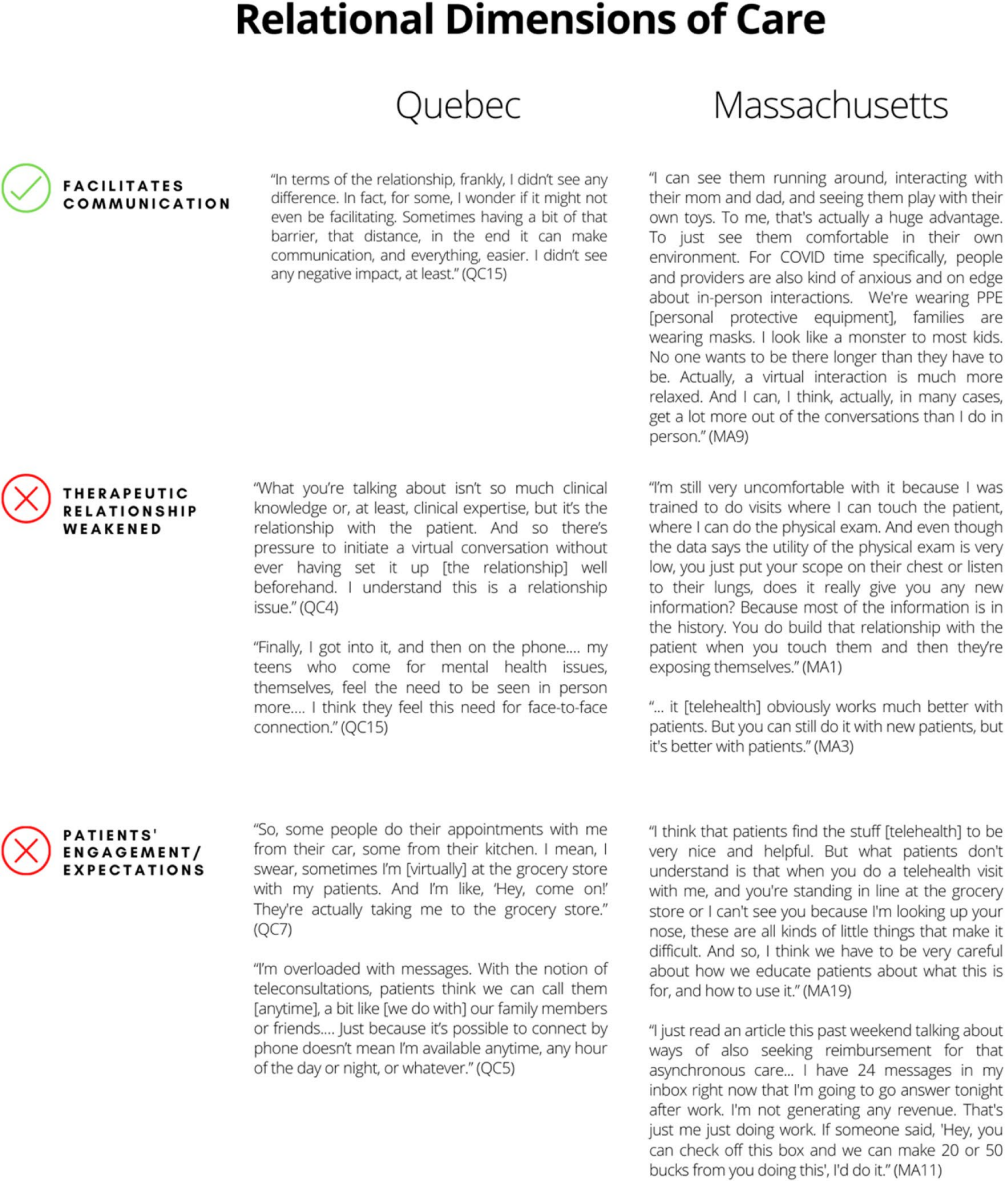
Positive and negative implications related to relational dimensions of care

In both contexts, the positive aspects of video consultations included patients’ comfort as well as providers’ ability to observe patients, their home environments, and their facial expressions. The fact that patients were usually in the comfort of their homes during telehealth appointments appeared to complement the advantages of physicians’ being able to observe patients in their home environments; this latter point was particularly noted by Massachusetts physicians. Moreover, seeing patients’ facial expressions was perceived to facilitate communication and contribute to developing or maintaining positive therapeutic relationships.

Negative aspects included the difficulty of maintaining the therapeutic relationship, limited patient engagement, and changes in patients’ expectations. Regardless of the telehealth modality (telephone or video), our results suggest physicians were concerned about the lack of direct human contact, which made it difficult to foster the therapeutic relationship. Although respondents acknowledged that face-to-face appointments and physical examinations were unnecessary to diagnose many conditions (see section on efficiency of care delivery), they viewed them as a major element of their practice and important for establishing trust and relationships with patients. This was reported to be particularly true for new patients or those in need of substantial psychosocial support.

Poor patient engagement was also reported as a telehealth challenge, seen in areas such as providers’ inability to reach patients (e.g., when patients would not answer the unidentified number) or reduced confidentiality of consultations (e.g., patients in public places or driving). For instance, respondents from both sites associated poor patient engagement with distractions caused by situations such as conducting their medical visits in inappropriate places (e.g., at a grocery store) or while driving. Finally, respondents from both sites were concerned that patients would expect more frequent consultations and communications because of their perceptions that telehealth made it relatively easy to contact their physician.

## Discussion

The perceptions of our respondents from Quebec and Massachusetts regarding the challenges and benefits of conducting telehealth during the COVID-19 pandemic were similar. Notably, the positive and negative perspectives on telehealth we found are consistent with the post COVID-19 literature [4, 11, 12, 18]. In line with the findings of a recent study in California, physicians in our study from both contexts believed telehealth offers opportunities for improving health care access, is well suited for caring for many medical conditions, and can enhance patient care in a variety of ways [18].

One significant difference was in the use of video consultations, which has spread more rapidly and widely in Massachusetts than in Quebec. This may be due to differences in incentives and reimbursement structures or in the availability of video telehealth training. Nevertheless, in comparing these two contexts, we identified challenges in delivering PHC services via telehealth: implementation issues, the need for physicians to develop new skills, impacts on the therapeutic relationship, and changes in interprofessional collaboration. These echo common challenges identified in recent qualitative studies on rapid implementation of telehealth that have reported on impacts on teamwork, access to care, technical problems, and relational issues that involve consultation, therapeutic relationships, confidentiality, and the ability to assess patients remotely [4, 11, 12].

### Implementation challenges

As the use of telehealth is increasing in parallel with continued use of face-to-face visits, it is essential to find strategies to ensure this care delivery modality is secure and equitable in both regular and crisis situations [4, 11, 13]. Our results highlight positive impacts for physicians, such as the comfort and flexibility of teleworking from home, flexibility in scheduling, improved availability for their patients, and the assurance of remuneration for telehealth appointments. Barriers to telehealth development before COVID-19 were due mainly to reimbursement limitations and health system organization concerns [2]. Pre-pandemic, physicians in Quebec’s public sector (70% of general practitioners and 82% of specialists in 2015) were remunerated on a fee-for-service basis [26, 27], but only specialist physicians were remunerated for telehealth [27–29], which mostly consisted of follow-up telephone calls. In the US, non-implementation of telehealth among physicians before the pandemic was due to concerns about reimbursement for telehealth services, medico-legal risks, potential inefficiencies, slow adoption of technological tools, and lack of telehealth training for healthcare professionals [30, 31].

Our results suggest that physicians appreciate that telehealth is now recognized as a formal care provision modality for which they can be remunerated. Of note, in both Quebec and Massachusetts, governments implemented temporary measures to remove this barrier during the pandemic and are looking to make these measures permanent. In Massachusetts, in March 2020, the governor issued an order requiring that private insurance cover all medically necessary telehealth video consultations and pay for them at the same rate as face-to-face consultations [31, 32]. In January 2021, the governor signed into a law a telehealth bill mandating payment parity for two years, giving the state and payers time to negotiate a long-term agreement on telehealth coverage. In Quebec, on March 16, 2020 [27, 33], the public insurance program was modified such that coverage of physician telehealth consultations (telephone and video) would be the same as for face-to-face visits [26, 27].

Clinicians’ prior reluctance to adopt telehealth stemmed, in part, from their negative perceptions of this tool [2, 34]. Because of its complexity, its significant changes to the way healthcare professionals practice, and perceptions that telehealth was not immediately effective, safe, or even common, many providers elected not to use it [2]. Our results show that, given their recent experience with telehealth, PHC providers from both contexts now have more nuanced views about the potential for reliable diagnoses via telehealth in various circumstances. In some situations, physical examination is not required and the patient’s history can be sufficient to make a diagnosis. On the other hand, diagnosing without a physical exam and visual information can be very challenging in some cases, with potential risk of error. A recent study showed that this inability to conduct direct physical examinations has been partly mitigated by involving patients in reporting their own data (e.g., their temperature) and by using video consultations to allow providers to observe patients’ general appearance and symptoms (e.g., breathing, coughing) [5]. Providers have also rediscovered the importance of taking a complete medical history and honing their observation skills to establish a diagnosis [11]. Our respondents also noted the need for guidelines to determine which cases can be most appropriately managed by telephone, video, or in person.

### Role change challenges

An additional difficulty in crisis situations, such as the COVID-19 pandemic, is the need to adopt new consultation methods quickly [2, 34]. In our study, the need for providers to modify roles and practices was clear. This is consistent with the results of a recent study [12] highlighting the compatibility or incompatibility of some professional roles with the provision of patient care through telehealth. Also, some professionals took on more tasks and had to adapt or assume new roles to support crisis management in their clinics, consistent with our findings, described above, about the need to replace nurses or administrative assistants in telephone triage during the pandemic.

With respect to the efficiency theme, training could be helpful to address the diagnostic and administrative challenges faced by providers. More experienced medical professionals seem to acquire telehealth skills more easily, which should be kept in mind when training future practitioners who will have had more experience with telehealth. In the present study, respondents perceived that the lack of training for specific skills related to using remote communication tools affected both the professional and relational aspects of care provision. Medical education is important in developing observational skills that need to be rediscovered and honed to establish a diagnosis remotely [11]. Delivering telehealth efficiently during a public health threat such as the COVID-19 pandemic requires that professionals be trained and equipped to use the various consultation tools, while also adapting to patients’ needs [35, 36]. However, after the crisis, reserving telehealth use for emergencies would be detrimental to its further advancement and to safe use [2]. Our respondents’ comments about the lack of guidelines on telehealth use mainly highlighted the negative impacts of not prioritizing the social and emotional aspects of care delivery along with the medical dimensions of care that are important in PHC.

### Therapeutic relationship challenges

A key role change for providers involves conducting a physical examination in the context of a virtual visit. Our respondents perceived that the physical examination is not only central to effective clinical practice, but also part of the physician’s role and a foundational element of the therapeutic relationship with the patient. They noted that this relationship was difficult to establish using only telehealth modalities, particularly when the physician had no previous relationship with the patient. Studies have also highlighted the risk that telehealth modalities can compromise the therapeutic relationship as well as continuity of care, aspects of care delivery that are central to clinical practice and profoundly significant for both patients and clinicians [4, 13, 14]. The patient–provider relationship is fundamental to effective treatment of mental, emotional, and behavioral health problems [14]. In the virtual care context, humanism should remain central [13]. Providers’ experiences during the current COVID-19 pandemic have underscored the necessity of developing social ties remotely for curative human relationships in addition to ensuring the safety and efficiency of care provision [14]. At the same time, fostering continuity of care and establishing therapeutic relationships with patients in a virtual care context implies developing new ways of initiating meaningful relationships through personal and situational practices [14].

Our results also suggest that technological barriers must not be underestimated, as they can affect patients’ access to care as well as physicians’ capacity to provide high-quality care. This is in line with findings from studies indicating that some patients and providers struggle with technological literacy and logistical barriers to participating in telehealth visits, especially with the different technologies available and/or the ways in which some medical practices have shifted technologies [4, 11, 12]. According to our results, these barriers can drive telehealth users to revert to telephone consultations (audio only), suggesting the need to address these technological issues.

### Interprofessional challenges

Elements perceived as problems in Quebec, such as the duplication of services, were seen differently in Massachusetts, where telehealth was perceived to be a tool that could be used to triage patients into those needing to be seen in person versus those whose visit could be conducted via telehealth. This use of telehealth, combined with effective interprofessional collaboration and a clear distribution of and complementarity in roles, may help improve telehealth efficiency while redistributing the workload equitably among professionals.

In line with another recent study [12], our results revealed personal impacts of telehealth that were positive, such as the sense of accomplishment gained from supporting colleagues and patients during the COVID-19 crisis, and others that were negative, including isolation, worry, and exhaustion. Our findings highlighting the importance of teamwork and of adapting to the transition to virtual care through constructive team meetings on safe care were consistent with prior findings related to this construct [12]. Finally, our findings regarding the negative impacts of telehealth on interprofessional work, including reported feelings of isolation due to the absence of interpersonal contacts and the loss of impromptu moments of socialization, such as in hallway discussions, also echoed that earlier study [12]. Given the necessity of interprofessional collaboration in health care delivery, our findings suggest adaptations must be made to facilitate such collaboration in telehealth, especially if telehealth care delivery options are to be sustained and more widely implemented.

### Strengths and limitations

One of the strengths of this study is that it draws on the perspectives of more than 40 PHC physicians in two different countries. Using a comparative approach allowed us to identify similarities and differences between the contexts, thereby increasing the credibility of our findings. This study also has limitations. One of these relates to the conditions of rapid change inherent in the context of COVID-19. We acknowledge that our results are based on the perceptions of participants at a particular moment in the health crisis and do not represent the general experience in PHC practice, a situation which is continuing to unfold. Subsequent research could periodically explore physicians’ and other stakeholders’ perspectives of telehealth in PHC practice to describe and understand how its impacts evolve over time. Future research on the implications of using telehealth over a more sustained period after the pandemic will be helpful to better understand the role of telehealth primary healthcare delivery. Also, future research to consider patients’ perspectives regarding telehealth and to track those perspectives over time would also provide valuable insight.

## Conclusion

The objective of this study was to explore the implications of conducting telehealth in PHC during the COVID-19 pandemic as reported by physicians in Quebec and Massachusetts. We conducted video interviews, and our thematic analysis revealed positive and negative implications of major issues such as access to care for patients, efficiency of care delivery, and professional and relational aspects of this care delivery modality. To ensure that telehealth care delivery meets the needs of both patients and providers, it will be critical to support the implementation of telehealth, provide guidelines and training to address professional challenges, and pay close attention to both technological barriers and human relationship needs. We believe that addressing these issues can help to mitigate barriers and facilitate the implementation of safe and effective virtual care.

## Supplementary Information

**Additional file 1.**

## Data Availability

The datasets used and/or analyzed for this study are available from the corresponding author on reasonable request.

## Abbreviations

AMA: American Medical Association
COVID-19: Coronavirus disease 2019
EMR: Electronic Medical Record
PHC: Primary healthcare
US: United States
